# 

**Published:** 1898-09

**Authors:** 


### CONTENTS.

ORIGINAL COMMUNICATIONS—

"Recurrent Gonorrhea." By Ferd. C. Valentine, M.D., New York ........ 433

The Serum Diagnosis of Typhoid Fever. By Michael Hoke, M.D., Atlanta, Ga. ........ 440

Some Abdominal Cases. By George Ben Johnston, M.D., Richmond, Va. ........ 444

Scott's Emulsion of Cod-Liver Oil in Eye and Laryngeal Troubles. By A. Bethune Patterson, M.D., Atlanta, Ga. ........ 451

NEW YORK LETTER ........ 454

CORRESPONDENCE ........ 459

EDITORIAL—

Medical Colleges and the American Medical Association ........ 465
The Patent on Antitoxin ........ 467
The American Medical Press ........ 468
Over-Production of Physicians ........ 472
The Olmsted Bill ........ 474
Notice to Subscribers ........ 475
Medical Examining Board in Georgia ........ 475

NEWS AND NOTES ........ 479

BOOK REVIEWS, PAMPHLETS, EXCHANGES—

Book Reviews ........ '88
Pamphlets and Exchanges ........ 491
Magazine Notes ........ 492

SELECTIONS AND ABSTRACTS ........ 493
MEDICAL ITEMS—Continued ........ x, xi, xii, xiii, xiv
ADVERTISERS' INDEX TO ADVERTISEMENTS ........ xvi

